# Lactic Acid Bacteria Bacteriocins: Safe and Effective Antimicrobial Agents

**DOI:** 10.3390/ijms26094124

**Published:** 2025-04-26

**Authors:** Xiaoyu Chen, Huili Bai, Weiliang Mo, Xunan Zheng, Hailan Chen, Yangyan Yin, Yuying Liao, Zhongwei Chen, Qingchi Shi, Zecheng Zuo, Zhengmin Liang, Hao Peng

**Affiliations:** 1 Guangxi Key Laboratory of Animal Breeding, Disease Control and Prevention, College of Animal Science and Technology, Guangxi University, Nanning 530004, China; cxy13766902977@163.com (X.C.); baihuili2020@163.com (H.B.); hlchen319@163.com (H.C.); yinyangyan@163.com (Y.Y.); 2 Guangxi Key Laboratory of Veterinary Biotechnology, Guangxi Veterinary Research Institute, Nanning 530001, China; liaoyuying@126.com (Y.L.); chen_zhong-wei@163.com (Z.C.); 3 Jilin Province Engineering Laboratory of Plant Genetic Improvement, College of Plant Science, Jilin University, Changchun 130062, China; mowl@jlu.edu.cn (W.M.); zhengxn21@mails.jlu.edu.cn (X.Z.); shiqc23@mails.jlu.edu.cn (Q.S.); zuozhecheng@jlu.edu.cn (Z.Z.)

**Keywords:** antimicrobial peptides, LAB bacteriocins, antimicrobial resistance, antimicrobial mechanism, food preservation, agricultural antimicrobials

## Abstract

Antibiotic-resistant bacteria are major contributors to food spoilage, animal diseases, and the emergence of multidrug-resistant (MDR) bacteria in healthcare, highlighting the urgent need for effective treatments. Bacteriocins produced by lactic acid bacteria (LAB) have gained attention for their non-toxic nature and strong antimicrobial properties. LAB-derived bacteriocins have been successfully applied in food preservation and are classified by the U.S. Food and Drug Administration (FDA) as ‘food-grade’ or ‘generally recognized as safe’ (GRAS). This review summarizes recent progress in the production, purification, and emerging applications of LAB bacteriocins. It emphasizes their versatility in food preservation, agriculture, and medicine, providing insights into their role in antimicrobial development and functional food innovation.

## 1. Introduction

Pathogenic bacteria present significant challenges to agriculture, healthcare, and the food industry [1]. These bacterial pathogens are responsible for millions of cases of food poisoning annually and some of them like rice bacterial blight can reduce crop yields by up to 50%, leading to billions of dollars in economic losses [2,3]. Particularly concerning is the threat of antibiotic-resistant bacteria, which account for over 700,000 deaths globally each year—a number that could escalate to 10 million deaths annually by 2050 if effective measures are not implemented [4]. Various strategies have been proposed to mitigate these bacterial threats, including improved hygiene practices, phage therapy, probiotics and prebiotics, antimicrobial peptides, and vaccination [5]. Among these alternatives, bacteriocins have emerged as promising candidates due to their natural safety, low potential for resistance development, and unique targeted antimicrobial capabilities [6,7]. In general, bacteriocins typically exhibit antimicrobial activity against species closely related to the producing strain, resulting in a relatively narrow spectrum of action. However, nisin, a bacteriocin approved by the FDA as “food-grade” or GRAS (Generally Recognized as Safe), demonstrates a broader antimicrobial spectrum, highlighting the potential versatility of certain bacteriocins.

Lactic acid bacteria (LAB) are a group of Gram-positive, non-spore-forming microorganisms characterized by their acid tolerance, heat resistance, and the absence of a noticeable odor. Morphologically, they can be rod-shaped (e.g., *Lactobacillus*) or spherical (e.g., *Lactococcus*) and are classified as facultative anaerobes [8]. The major genera of LAB include *Lactiplantibacillus*, *Latilactobacillus*, *Levilactobacillus*, *Lactococcus*, etc. [9]. LAB bacteriocins, which are commonly found in fermented foods, are known for their prolific production. They are non-toxic, exhibit a low propensity for inducing resistance, and possess potent antimicrobial activity [10]. The antimicrobial action of LAB bacteriocins involves disrupting cell membrane integrity and inhibiting protein and nucleic acid synthesis, making the development of resistance challenging [11,12]. LAB bacteriocins can retain their activity for extended periods under freeze-drying or refrigeration conditions. Many bacteriocins have narrow activity spectra, targeting primarily strains closely related to their producers. In contrast, some exhibit broad-spectrum antimicrobial activity against diverse genera. The regulation of bacteriocin production is often complex and can be influenced by various environmental factors, including pH, temperature, and the composition of the growth medium [13]. As proteinaceous compounds, bacteriocins are susceptible to enzymatic degradation after absorption [14]. Magnusson et al. isolated over 1200 strains of LAB from various environments. Some of the strains showed strong to moderate antimicrobial activity against *Aspergillus fumigatus*, *Aspergillus nidulans*, *Penicillium commune*, etc. And antifungal cyclic dipeptides produced by lactic acid bacteria were identified [15]. These characteristics highlight their potential as viable alternatives to commercial antibiotics and chemical preservatives. The application potential of LAB bacteriocins spans multiple fields. In the food industry, they serve as natural preservatives, effectively inhibiting common foodborne pathogens such as *Listeria monocytogenes*, *Salmonella Typhimurium*, and *Staphylococcus aureus* [16]. By enhancing food safety while reducing reliance on chemical preservatives, LAB bacteriocins align with consumer demand for natural and clean-label products [17]. Beyond food preservation, LAB bacteriocins also demonstrate potential as eco-friendly biocontrol agents in agriculture, suppressing plant pathogens and promoting sustainable farming practices [18]. In the biomedical field, LAB bacteriocins show promise as antibiotic alternatives for combating multidrug-resistant bacteria, highlighting their critical role in addressing the global antibiotic resistance crisis [6].

This review provides an overview of the fundamental types of LAB bacteriocins and the strategies employed for their discovery, isolation, purification, and identification. It also summarizes the synthesis, structural forms, and antibacterial activities of bacteriocins. Particular emphasis is placed on recent advances in production strategies, purification techniques, and emerging applications of bacteriocins.

With their unique properties and broad application potential, LAB bacteriocins are poised to become essential tools in enhancing food safety, promoting green agriculture, and driving biomedical innovations. Supported by technological advancements, favorable policies, and international collaboration, LAB bacteriocins are expected to make significant contributions to achieving global sustainability goals.

## 2. Fundamental Characteristics and Classification of Bacteriocins

### 2.1. Key Developments of Bacteriocins

Bacteriocins, natural antimicrobial peptides synthesized by bacteria during the logarithmic growth phase, are garnering attention for their safety, efficacy, and biodegradability. These small molecules play a crucial role in microbial communities by inhibiting the growth of harmful bacteria, thereby providing a competitive advantage to the producing bacteria within their environment [19,20]. Research on bacteriocins began in 1925 when Fath first discovered colicin V in the fermented broth cultures of *Escherichia coli* [21]. In 1928, Rogers identified a substance produced by *Lactococcus lactis* with broad-spectrum inhibitory activity against Gram-positive bacteria, naming it Nisin A. Nisin A became the first bacteriocin widely used for food preservation [22,23,24]. In 1951, researchers identified Subtilin, produced by *Bacillus subtilis*, which was primarily used to inhibit pathogenic and spoilage bacteria in food [25]. In 1956, bacteriocins were first defined as proteins with antimicrobial activity produced by bacteria, laying the foundation for subsequent research and classification [23].

**Figure 1:**
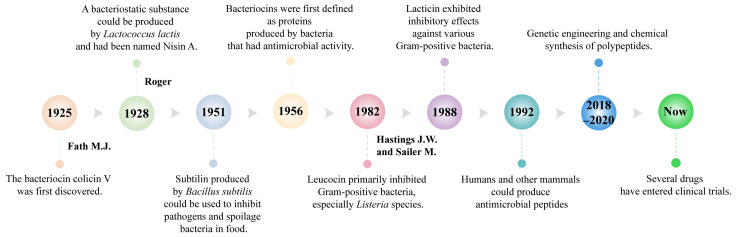
Key milestones of antimicrobial peptides.

Over the years, researchers have discovered an increasing number of bacteriocins (Figure 1). Qiao et al. found that BMP32r, a type of bacteriocin, disrupts mature biofilms by killing bacteria in the biofilm and its effectiveness in killing *Listeria monocytogenes* persisters makes it a promising potential anti-biofilm agent against *Listeria monocytogenes* [26]. In 1992, Lacticin 481, produced by *Lactococcus lactis*, was identified as an inhibitor of various Gram-positive bacteria [27]. In 2016, Zipperer et al. found that lugdunin produced by *Staphylococcus. lugdunensis* had a broad spectrum of biological activity against Gram-positive bacteria, including methicillin-resistant *Staphylococcus aureus*, vancomycin, and glycopeptide-intermediate-resistant *Staphylococcus aureus* strains [28]. Li et al. discovered that sublancin produced by *Bacillus subtilis* can act directly on the host, selectively triggering the innate immune response and modulating the structural and functional composition of the microbiota to protect mice from pathogens [29]. Khorshidian et al. discovered that pediocin produced by *Pediococcus* could be effective against many Gram-positive bacteria, particularly *Listeria*, and is commonly used for preserving meat and meat products [30].

Since Klaenhammer first proposed a classification method for bacteriocins in 1936, advancements in high-throughput technologies have significantly progressed the field [31]. Detailed investigations into the structure, function, and mechanisms of bacteriocins have led to the ongoing identification and characterization of new variants, resulting in the evolution of the initial classification scheme over time [8,32]. Currently, after an extensive literature review, four main categories of bacteriocins have been identified and confirmed [33,34]. The following will be a detailed discussion of the classification and basic characteristics of LAB bacteriocins, and will not be further expanded here.

### 2.2. Fundamental Characteristics of LAB Bacteriocins

**Figure 2:**
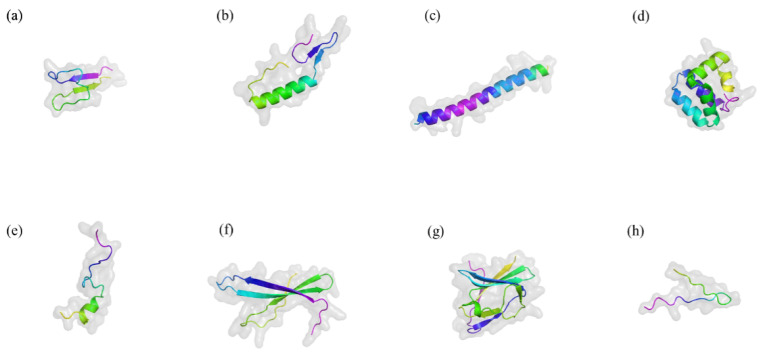
Typical three-dimensional structure of several bacteriocins. Drawn by Alphold (https://alphafoldserver.com/ accessed on 20 October 2024). (**a**) Nisin A. (**b**) Pediocin PA-1. (**c**) Lactococcin G. (**d**) Enterocin AS-48. (**e**) Bacteriocin 31. (**f**) Helveticin J-11. (**g**) Enterolysin A. (**h**) MccJ25. The figure illustrates the classic three-dimensional structures of antimicrobial peptides, including α-helix, β-sheet, extended structure, and loop and extended regions. These structures interact with bacterial membranes, penetrating and disrupting their integrity, thereby exerting antimicrobial effects.

LAB, first reported in 1982, have been isolated from a variety of environments, including fermented cucumbers, silage, bioethanol production plants, dairy products, and brewing wort [35]. They are Gram-positive, non-sporulating microorganisms that produce bacteriocins—proteinaceous or peptide compounds with significant antimicrobial activity. They are classified as obligate homofermentative, obligate heterofermentative and facultative heterofermentative. LAB bacteriocins are known for their remarkable thermal stability and tolerance to acidic and alkaline conditions, allowing them to remain active in diverse environments [36]. Notably, the antimicrobial activity of LAB bacteriocins exhibits significant specificity [8]. Some bacteriocins target closely related bacterial strains with high precision, while others demonstrate a broader spectrum, effectively inhibiting pathogens across multiple genera [16]. Moreover, LAB are easy to culture and store, offering benefits such as regulating gut microbiota, modulating the immune system, antagonizing pathogens, and promoting animal growth [37]. They not only improve gut microbiota but also positively impact food production and fermentation processes [10,38]. They have a DNA G+C content of less than 50 mol% and can produce a wide range of digestive enzymes and small bioactive compounds under both homolactic and heterolactic fermentation conditions [39]. Figure 2 illustrates the structures of several representative bacteriocins [40].

### 2.3. Classification of LAB Bacteriocins

Since Klaenhammer introduced the first classification of LAB bacteriocins in 1993 [31], numerous bacteriocins have been isolated from LAB. Although LAB bacteriocins fit the definition of antimicrobial peptides, the newly identified RiPP (ribosomally synthesized and post-translationally modified peptides, a class of bioactive peptides produced through ribosomal synthesis of precursor peptides followed by enzymatic post-translational modifications) subgroups and their activities deviate from traditional bacteriocin classifications. Class IV bacteriocins are complex cyclic peptides composed of lipids and carbohydrates [41]. However, to date, no LAB bacteriocins have clearly demonstrated these properties, leaving this class relatively underexplored [42]. Based on the revised suggestions proposed by Cotter et al. in 2005, LAB bacteriocins have been simplified into two categories [42]: Class I (modified bacteriocins) and Class II (unmodified bacteriocins), as distinguished in the revised classification. The original Class III was renamed “lysozymes,” and Class IV was eliminated. While this section focuses on bacteriocins isolated from LAB, the classification scheme is also applicable to known antimicrobial peptides isolated from other ecological environments. Based on molecular weight, chemical structure, thermal stability, and post-translational modifications, LAB bacteriocins are primarily classified into three categories [33,43,44].

Class I (less than 10 kDa): Class I bacteriocins are small peptides that undergo post-translational modifications during biosynthesis. These bacteriocins feature a leader peptide, which plays a role in recognition, transport, and maintaining the activity of the core peptide, and is subsequently fused with the core peptide [45]. Medema et al. provided key characteristics for the systematic definition of Class I bacteriocins. Lanthipeptides, the most extensively studied within Class I, contain special amino acids and typically include residues like lanthionine, heterocyclic structures, head-to-tail cyclization, and glycosylation [46,47]. The genes involved in the maturation of lanthipeptides are typically located within the same operon. Type I (Lan-BC modification) and Type II (LanM-modified) are considered antibiotics [48]. Types III and IV lack known antimicrobial activity and are not further discussed here.

Class II (greater than 10 kDa): Class II bacteriocins are non-modified, heat-stable, non-thiol peptides. Most of them are cationic peptides, and their antimicrobial activity is primarily exerted by enhancing membrane permeability [49,50]. They are further classified into four subclasses based on their structural features. Class IIa bacteriocins, characterized by a double glycine-type signal sequence, are exemplified by Pediocin PA-1, which shows strong inhibitory activity against *Listeria monocytogenes* in a model gut environment [51]. Class IIb bacteriocins consist of two peptides that synergistically enhance activity through disulfide bonds formed by cysteine residues, such as Lactococcin G [52]. Class IIc bacteriocins are cyclic peptides with covalently bonded N- and C-termini, typically exceeding 30 kDa in molecular weight and exhibiting a narrow antimicrobial spectrum [53]. Class IId bacteriocins are linear, single-chain peptides that may contain thioether bonds or lack cysteine residues, often displaying broad-spectrum antimicrobial activity [54].

Class III bacteriocins are high-molecular-weight (30–80 kDa), heat-sensitive proteins primarily derived from lactic acid bacteria and other Gram-negative bacteria [55]. Their heat sensitivity limits their application in food preservation.

In 2016, researchers analyzed 238 complete *Lactobacillus* genomes in a database, revealing that the numbers of gene clusters encoding presumed Class I, II, and III bacteriocins were 137, 514, and 97, respectively. This study found that Class II bacteriocins are the most abundant antimicrobial peptides in *Lactobacillus* [8]. According to bacterial taxonomy, there are six genera of LAB, with their names and ecological origins presented in Table 1.

Most bacteriocins studied in LAB belong to Class I or Class II due to their heat stability, which makes them particularly suitable for food preservation. These bacteriocins typically exhibit high selectivity, targeting specific bacterial species [56,57,58,59].

**Table 1:** Origins and representative bacteriocins of LAB [60].

Genus	Ecological Origin	Examples
*Lactococcus*	Fermented food, dairy products	Nisin A, Lactococcin Z
*Lactobacillus*	Fermented foods, plants	Bacteriocin ST69BZ, Sakacins D98a
*Streptococcus*	Animal gastrointestinal tract	Lactostrepcin, Sb15
*Pediococcus*	Fermented foods, plants	Pediocin PA-1, Pediocin ST18
*Carnobacterium*	Refrigerated food, ocean	Piscicolin 61, Divergicin 750
*Enterococcus*	Human and animal gastrointestinal tract	Enterocin A, Cytolysin

## 3. Biosynthesis of Bacteriocins

Bacteriocins are ribosomally synthesized peptide compounds that are initially biologically inactive and require post-translational modifications to become functionally active [61]. The genes encoding bacteriocins are typically clustered within operons, which may be located on chromosomes, plasmids, or sometimes inserted into chromosomes via transposons [61]. The biosynthetic pathway of bacteriocins is relatively conserved, encompassing the synthesis of precursor bacteriocins, cleavage of the leader sequence at specific processing sites, and secretion of the precursors into the extracellular environment [62]. During this process, the operons include multiple functional modules that regulate the synthesis, processing, and secretion of bacteriocins [63,64]. Prior to secretion, precursor bacteriocins undergo further modifications, such as the formation of thioether cross-links (e.g., lanthionine or methyllanthionine) through the degradation of serine or threonine residues, or the addition of cysteine residues to unsaturated amino acids, enhancing their structural stability and biological activity.

### 3.1. Key Components and Signal Transduction in Bacteriocin Synthesis

The synthesis of bacteriocins involves five main types of genes. Structural genes encode the precursor proteins of bacteriocins, typically carrying an N-terminal leader sequence (such as double glycine-type or signal peptide-type) [65]. The two conserved glycine residues at the C-terminus are recognized by ABC transporters, which cleave the leader sequence and secrete the mature bacteriocin. During this process, the signal peptide sequence plays a crucial role in the processing and secretion of bacteriocins via the general secretory pathway [66]. Immunity genes encode small proteins comprising 51–154 amino acids, which protect the producing bacteria from the toxic effects of their own bacteriocins [67]. Transport and processing genes encode proteins responsible for the processing, transport, and secretion of precursor bacteriocins, ensuring their proper release [68]. Modification genes encode enzymes that carry out post-translational modifications, converting precursor bacteriocins into their active, mature forms [69]. Lastly, regulatory genes control the expression of bacteriocin synthesis genes by regulating signaling pathways, thereby enabling efficient bacteriocin production under appropriate conditions [70].

The production and secretion of bacteriocins are regulated by a signal transduction system comprising three key components: induction peptide, response regulatory protein, and sensor histidine protein kinase (HPK). The induction peptide is a small cationic molecule that forms an amphipathic α-helix and serves as a signaling molecule for the regulatory system, also referred to as a “quorum-sensing” signal [71]. It plays a crucial role in controlling the synthesis of specific bacteriocins. The response regulatory protein transmits the signal, acting as a key mediator in the signal transduction process, while HPK detects the signal and initiates the corresponding regulatory processes [72,73].

Two primary models have been proposed to explain the induction mechanism for bacteriocin synthesis. The first model suggests that induction peptides are continuously produced in small quantities during bacterial growth and gradually accumulate. When their concentration reaches a specific threshold, the expression of bacteriocin-related genes is triggered [74]. The second model proposes that the production of induction peptides can temporarily increase under certain environmental conditions. Once the concentration surpasses the self-induction threshold, the expression of bacteriocin gene clusters is activated, accelerating bacteriocin synthesis [74].

### 3.2. Characterization of Synthesis of Different Classes of Bacteriocins

The biosynthesis of bacteriocins involves the ribosomal synthesis of precursor proteins, followed by various post-translational modifications, cleavage, and maturation processes [75]. These processes vary across the four classes of LAB bacteriocins, resulting in diverse structures and modes of action.

Class I bacteriocins, such as Nisin and Lacticin 3147, undergo post-translational modifications involving dehydratases and cyclases. Nisin, produced by *Lactococcus lactis*, was discovered and commercialized in the 1950s [76]. The precursor protein and its functional domains (NisB, NisC, and NisP) are synthesized ribosomally. The substrate NisA undergoes dehydration of serine and threonine residues by the dehydratase NisB and the cyclase NisC. This process results in the formation of dehydrated amino acids, which subsequently interact with cysteine thiols to form thioether bridges, completing the cyclization. Specific proteases, such as NisP, then cleave the leader sequence, releasing the mature bacteriocin [77]. In addition to Nisin, other lantibiotics, such as Lacticin 481, undergo similar modifications [78]. Non-lantibiotic Class I bacteriocins do not undergo extensive modifications. They possess mature head-to-tail structures, and their cyclic conformation confers stability, allowing them to withstand various environmental stresses [79].

Class II bacteriocins are divided into four subclasses, each with distinct formation mechanisms. Generally, the genes associated with Class II bacteriocins include those encoding precursor proteins, immunity proteins, transporter proteins, and membrane-bound auxiliary proteins [80]. The biosynthesis process begins with the synthesis of precursor proteins containing mature antimicrobial peptide sequences and leader peptides. Leader peptides, which are recognized and cleaved by signal peptidases, are crucial for the proper folding and maturation of antimicrobial peptides. The cleaved precursor proteins undergo further processing by immunity proteins, typically proteases, to release the mature antimicrobial peptides [81].

Class III bacteriocins, which include lysozymes (IIIa) and non-lysozymes (IIIb), have different formation mechanisms compared to Classes I and II [43]. Their synthesis is tightly regulated at the transcriptional level, involving specific promoters and transcription factors. These high-molecular-weight, heat-labile proteins undergo post-translational modifications, such as folding, assembly, or glycosylation, before being released into the environment.

Class IV bacteriocins, also known as complex bacteriocins, contain lipid or carbohydrate groups [82]. During their synthesis, precursor proteins bind with these lipid or carbohydrate groups, resulting in structures with a high content of hydrophobic residues. Specific proteases then cleave the leader sequence to release the mature bacteriocin. These modifications can increase hydrophobicity and stability, thereby enhancing the bacteriocins’ effectiveness in diverse environmental conditions. These complex bacteriocins serve as potent antimicrobials, particularly in nutrient-rich environments where competition is high [82].

## 4. Antimicrobial Mechanisms of LAB Bacteriocins

Antibiotics have traditionally been the cornerstone of bacterial infection treatment. However, their widespread use has led to significant issues with bacterial resistance. Unlike antibiotics, most bacteriocins exhibit a narrower spectrum of activity but are valued for their excellent stability, structural diversity, and low toxicity. Additionally, their specific antimicrobial mechanisms and biosynthesis processes reduce the likelihood of resistance development.

**Figure 3:**
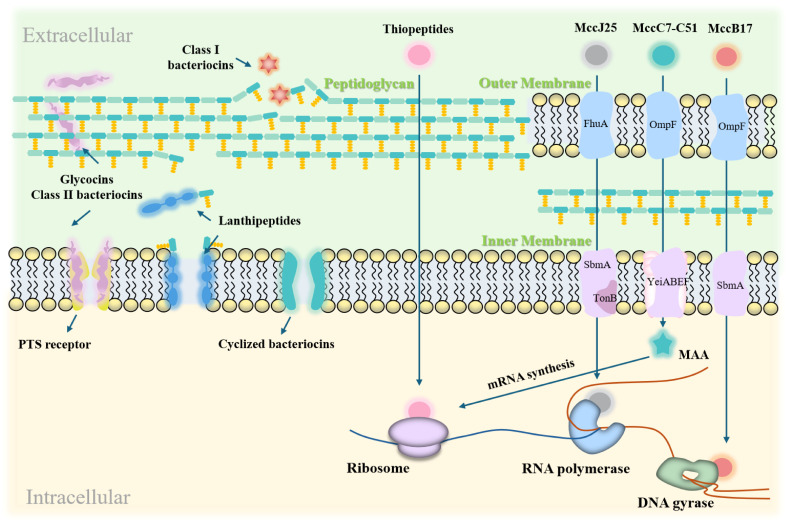
Action modes of LAB bacteriocins on Gram-positive and Gram-negative bacterial targets. LAB bacteriocins exert their effects by disrupting cell membranes, forming pores, and interfering with gene expression and protein synthesis. In Gram-positive bacteria, they inhibit growth by forming membrane pores and blocking peptidoglycan synthesis. In Gram-negative bacteria, they inhibit growth by damaging cell membranes and disrupting cellular functions.

LAB bacteriocins are natural antimicrobial substances that have garnered significant attention in both the food industry and the medical field [83,84]. Their primary mechanism of action involves disrupting bacterial cell membranes, leading to bacterial cell death [85]. Figure 3 illustrates the modes of action of LAB bacteriocins against Gram-positive and Gram-negative targets.

### 4.1. Mechanisms of Cell Membrane Disruption

As illustrated below, the most common mechanism by which bacteriocins exert their effects is through specific binding to receptors on the bacterial cell membrane. This binding leads to structural disruption of the membrane. This disruption creates pores in the membrane, causing leakage of cellular contents and ultimately leading to cell death. Bacteriocins achieve this through the following mechanisms:

Pore Formation: Class II bacteriocins create pores in the cytoplasmic membrane, leading to increased permeability [81]. The net charge and hydrophobic properties are the primary factors driving the binding of bacteriocins to bacterial cell membranes. Cationic bacteriocins interact with the anionic bacterial cell membrane, promoting the aggregation of bacteriocins on its surface. Hydrophilic groups of the bacteriocins embed within the lipid molecules, while the hydrophobic groups face the external environment. This orientation leads to the formation of ion channels, known as pores and gates [86]. For example, bacteriocin BM1122, produced by *Lentilactobacillus crustorum* MN047, creates channels in the cell membrane, increasing permeability. This causes leakage of cytoplasmic components, ultimately leading to bacterial death [87]. Additionally, other Class II bacteriocins like nisin A and pediocin PA-1 bound to the mannose phosphotransferase system (Man-PTS) on the cell membrane, leading to pore formation [81].

Increased Membrane Permeability: The action mechanism of Class I bacteriocins, such as lanthipeptides, increases membrane permeability. These bacteriocins possess hydrophobic and positively charged surfaces that interact electrostatically with negatively charged phospholipids. Ion channels cause cell membrane rupture or leakage of intracellular substances, increasing membrane permeability and disrupting normal metabolic functions. This membrane disruption ultimately leads to irreversible cellular collapse and bacterial lethality. For instance, the novel bacteriocin LSB1 from *Lactiplantibacillus* disrupts the physical barrier of *Streptococcus mutans* cells and disturbs the balance of intracellular and extracellular substances in vitro [88].

Compared to traditional antibiotics, LAB bacteriocins are generally less prone to inducing resistance. Nonetheless, bacteriocins that target bacterial nucleic acids may still carry a potential risk of resistance development. In vivo studies have indicated that resistance mechanisms in *Staphylococcus aureus* to nisin often involve alterations in membrane lipid composition or the activation of efflux pumps [89]. Moreover, environmental stressors, such as triclosan (TCS), have been directly linked to increased resistance, with TCS resistance being associated with heightened resistance to other antibiotics [90]. To mitigate these risks, it is crucial to explore the combination of LAB bacteriocins with other antimicrobial agents and further investigate the genetic mechanisms underlying resistance, aiming to reduce the likelihood of LAB bacteriocin resistance.

### 4.2. Interference with Gene Expression and Protein Synthesis

Bacteriocins can also inhibit bacterial growth by interfering with gene expression and protein synthesis through specific mechanisms:

Disruption of DNA and RNA replication: Bacteriocins block the transfer of genetic information, thereby inhibiting gene expression [91]. The exceptional activity of Microcin J25 (MccJ25) against pathogenic enterotoxigenic *Escherichia coli* (ETEC) is attributed to its ability to permeate bacterial membranes and its strong affinity for the secondary channel of RNA polymerase, which inhibits the transcription process. MccJ25 exhibits high endotoxin-neutralizing activity both in vitro and in vivo, and significantly suppresses the secretion and expression of pro-inflammatory factors. Importantly, MccJ25 does not exhibit a significant mutation rate, reducing the likelihood of developing antibiotic resistance [92].

Inhibition of Protein Synthesis: Bacteriocins can target components involved in protein synthesis, disrupting bacterial protein production. For instance, the bacteriocin MccC7-C51 inhibits aspartyl-tRNA synthetase, thereby blocking mRNA synthesis [93]. Bacteriocin MccB17 interferes with DNA replication by inhibiting DNA gyrase-mediated supercoiling [94]. Additionally, bacteriocins can alter cell membrane permeability, inhibit cellular respiration and spore germination, and disrupt intracellular enzyme activity [43]. These effects interfere with normal bacterial functions and ultimately lead to cell death.

### 4.3. Differential Mechanisms of Action Against Gram-Positive and Gram-Negative Bacteria

Bacteriocins employ different mechanisms of action against Gram-positive and Gram-negative bacteria:

Gram-Positive Bacteria: Bacteriocins primarily inhibit these bacteria by forming membrane pores and blocking peptidoglycan synthesis. For example, Nisin binds to lipid II molecules, inhibiting peptidoglycan synthesis and forming pores in the cell membrane, which leads to cell lysis in vitro [95]. Class III bacteriocins, including lysozyme-like bacteriocins, are crucial for degrading bacterial cell walls by hydrolyzing β-1,4-glycosidic bonds in peptidoglycan, making them particularly effective against Gram-positive bacteria [43].

Gram-Negative Bacteria: Bacteriocins inhibit these bacteria by disrupting the functions of both the outer and inner membranes. For example, pediocin alters outer membrane permeability and interferes with protein synthesis, thereby inhibiting bacterial growth [30].

## 5. Production of LAB Bacteriocins

The production and optimization of LAB bacteriocins involve meticulous processes including isolation, purification, and identification, as well as strategies to enhance production efficiency [96]. These antimicrobial peptides are derived from various natural sources and undergo rigorous testing and refinement to achieve high purity and functional characterization. Advanced techniques, such as chromatography and gene sequencing, are employed to analyze the structures and properties of these antimicrobial peptides [97]. Additionally, innovative approaches such as heterologous expression, optimization of cultivation conditions, and genetic modifications are pivotal for maximizing bacteriocin yield and effectiveness [96,98].

### 5.1. Challenges in the Production of LAB Bacteriocins

The isolation, purification, and characterization of bacteriocins produced by LAB is a complex process requiring a series of advanced techniques to ensure high purity and functional analysis. Several challenges arise during the separation and purification stages. First, fermentation broths contain numerous secondary metabolites, and the actual concentration of bacteriocins is typically low. This not only increases the difficulty of separation but also potentially compromises detection accuracy during characterization. Second, compared to general proteins, LAB bacteriocins have relatively small molecular weights, making their sizes similar to certain peptide fragments present in the culture medium, thereby adding to the complexity of the purification process [99]. Common methods for the isolation and purification of LAB bacteriocins include ammonium sulfate precipitation, membrane filtration, and various chromatographic techniques [100]. Among these, ammonium sulfate precipitation is the most widely used salting-out method [101]. However, the ammonium sulfate precipitation method has limitations, as high salt concentrations can cause bacteriocin denaturation, thereby reducing its antimicrobial activity [102]. Ion-exchange chromatography exploits the positively charged nature of bacteriocins under specific pH conditions to separate proteins with different properties. During the purification of LAB bacteriocins, NaCl gradients are typically employed for elution to isolate the target bacteriocins effectively [27,102]. For example, Callewaert et al. employed a strong cation exchanger to directly isolate amylovorin L471, a bacteriocin from *Lactobacillus amylovorus* DCE 471, from the fermentation medium [103]. Membrane filtration technology, on the other hand, separates target peptides based on molecular weight cutoffs. Fricourt et al. successfully isolated the bacteriocin Plantaricin F from the fermentation supernatant of Lactiplantibacillus BF001 using ultrafiltration with a molecular weight cutoff of 10,000 Da [104].

It is established that the main method of bacteriocin research is PCR analysis, which makes it possible to quickly and easily identify the presence of bacteriocin encoding genes [105]. At the protein level, SDS-PAGE is a commonly employed analytical method used to evaluate the molecular weight and purity of bacteriocins. Zamfir et al. employed liquid chromatography to determine the amino acid sequence of a bacteriocin from *Acidophilus* IBB 801 [106]. Zafar et al. used the SDS-PAGE technique to characterize a bacteriocin produced by a strain of *Bifidobacterium* [107]. In addition, mass spectrometry techniques, such as LC-MS/MS, serve as standard methods for confirming molecular structure and purity, offering detailed information on molecular weight and composition [108]. More advanced structural analysis techniques further enhance the understanding of bacteriocin molecular structures. For example, nuclear magnetic resonance (NMR) spectroscopy enables precise molecular structure determination, while infrared (IR) spectroscopy identifies key functional groups within the molecule [109,110]. In addition to experimental techniques, bioinformatics and genomic tools have played a crucial role in the discovery and study of bacteriocins. Genomic mining methods allow for the rapid identification of new bacteriocin gene clusters. For instance, researchers have discovered novel bacteriocin gene clusters, including lactone peptides, in 224 species of rumen bacteria and 5 species of rumen archaea [111]. These tools not only accelerate the discovery of bacteriocins but also provide a foundation for functional modification and performance optimization [91,97,112].

While these techniques significantly advance bacteriocin research, challenges remain in achieving cost-efficient and scalable production. High-purity purification methods, such as RP-HPLC, are resource-intensive and may not be suitable for industrial-scale applications. Future efforts should focus on developing sustainable and cost-effective methods to bridge laboratory feasibility and practical implementation.

### 5.2. Strategies for Enhancing Production Efficiency

Researchers have employed various strategies to enhance the efficiency of bacteriocin production. As shown in Table 2, these approaches include heterologous expression, solid-phase synthesis, chemical or physical mutagenesis, optimization of production conditions, microbial co-culture techniques, gene editing, and computational modeling. Traditional culturing methods, although effective, often result in low yields and cumbersome extraction processes [113], prompting researchers to develop various strategies to enhance bacteriocin production efficiency. Heterologous expression is a common method in which bacteriocin genes are cloned into different host organisms to significantly increase yields. For example, Sommer et al. achieved the extracellular expression of Colicin E1 and Cloacin DF13 bacteriocins by co-expressing bacteriocin release proteins (BRPs) [114]. Jiménez et al. expressed Enterocin A in *Komagataella phaffii* and *Kluyveromyces lactis* [115]. Meanwhile, Li et al. heterologously expressed the hybrid peptide EF-1 in *Komagataella phaffii* cells [116]. Hayward et al. used high-throughput screening to discover and express the Nek2 cancer inhibitor from a bacteriocin associated with a Toxoplasma-like protozoan [117]. Chemical reagents such as 40% ammonium sulfate and chloroform/methanol are used for the crude extraction of bacteriocin proteins, followed by reversed-phase high-performance liquid chromatography (RP-HPLC) for purifying antimicrobial peptides [53]. Jiménez et al. also cloned the enterocin A gene into *Latilactobacillus sakei* Lb 790 for expression [118]. Solid-phase peptide synthesis (SPPS) is used to synthesize specific bacteriocin peptides. Additionally, chemical or physical mutagenesis is used to modify production strains, which enhances bacteriocin yield [119,120].

Heterologous expression has been widely utilized for the large-scale production of antimicrobial peptides. However, this process involves significant costs associated with the development of expression systems, culture maintenance, and downstream processing. For instance, the commonly used *Komagataella phaffii* expression system requires a substantial amount of carbon sources [116]. Similarly, advanced purification techniques such as RP-HPLC and affinity chromatography can achieve excellent purity. However, the high costs of chromatography materials and solvents significantly limit their feasibility for large-scale industrial production [107]. To address these challenges, researchers are actively exploring innovative approaches. Optimizing production conditions is crucial for increasing bacteriocin yield. Factors such as medium composition, pH, temperature, and microbial growth kinetics significantly impact bacteriocin biosynthesis [121]. For example, Dündar et al. improved bacteriocin extraction efficiency through pH-mediated cell adsorption [122]. Pediocin production is influenced by a number of nutritional parameters. Anastasiadou et al. found that glucose was the best carbon source for pediocin production in *Lactococcus lactis*, while glycerol was the strongest inhibitor of pediocin production [123]. Researchers also optimized fermentation technology by controlling factors such as oxygen supply, temperature, and pH. These adjustments increased bacteriocin yield and activity [124]. *Latilactobacillus curvatus/Pediococcus acidilactici* produced the highest BLIS (Bacteriocin-Like Inhibitory Substances, a protein or peptide substance produced by a bacterium that has antimicrobial activity) at a growth temperature of 28 °C and pH 5, and the optimal conditions for BLIS production by *Latilactobacillus sakei* were 24 °C and pH 6.5. BLIS production by *Latilactobacillus curvatus*/*Pediococcus acidilactici* bacteria was strongly influenced by carbon and nitrogen sources [125]. Qiao et al. increased the production of *Lactococcus lactis* by producing bacterial cellulose membranes with different properties and using a co-culture approach [126]. Castro et al. assessed the effect of several factors on BLIS production and found that BLIS produced by the LAB strain was strongly influenced by NaCl concentration and the presence of surfactant [127]. Microbial co-culture techniques are employed to enhance bacteriocin production. For instance, researchers established a co-culture strategy involving bacterial cellulose (BC) and *Lactococcus lactis*, which significantly increased lacticin 3147 yield and activity to 6260 IU/mL [128]. Gene editing technologies, such as CRISPR-Cas9, are employed to modify lactic acid bacteria, thereby enhancing bacteriocin production capacity. These technologies are currently applied in the production of fermented foods and nutritional products [129]. Precise gene modifications enable researchers to optimize metabolic pathways in lactic acid bacteria, directing more precursor substances toward bacteriocin synthesis [130]. This approach increases synthesis efficiency [131]. Additionally, computational modeling and systems biology approaches offer significant potential for optimizing bacteriocin production strategies. Computer models simulate bacterial growth and metabolism, while molecular dynamics (MD) simulations are used to verify structures and interactions [132,133]. Researchers employed systems biology to construct and screen a semi-library of Phyper-spank variants, gaining insights into bacterial metabolic networks. This work provides theoretical support for optimizing bacteriocin production [134]. These strategies not only significantly increase bacteriocin yield and purity but also enhance efficiency and cost control in the production process.

**Table 2:** Strategies for enhancing production of LAB bacteriocins.

Category	Strategies and Examples	References
Heterologous Expression	Expression in *Pichia*—*Kluyveromyces*	[118,124]
Solid-Phase Peptide Synthesis (SPPS)	Synthesis of specific bacteriocin peptides	[119]
Mutagenesis	Chemical or physical modification of production strains	[120]
Production Conditions	Control of pH, temperature, and oxygen during fermentation	[121,124]
Microbial Co-culture Techniques	Co-culture strategies to enhance bacteriocin production	[126]
Gene Editing (CRISPR-Cas9)	Modification of LAB to enhance bacteriocin production capacity	[128,130]
Computational Modeling	Simulation of bacterial growth and metabolism	[131,132]
Systems Biology Approaches	Human and animal gastrointestinal tract	[133]

## 6. Applications of LAB Bacteriocins in Various Industries

**Figure 4:**
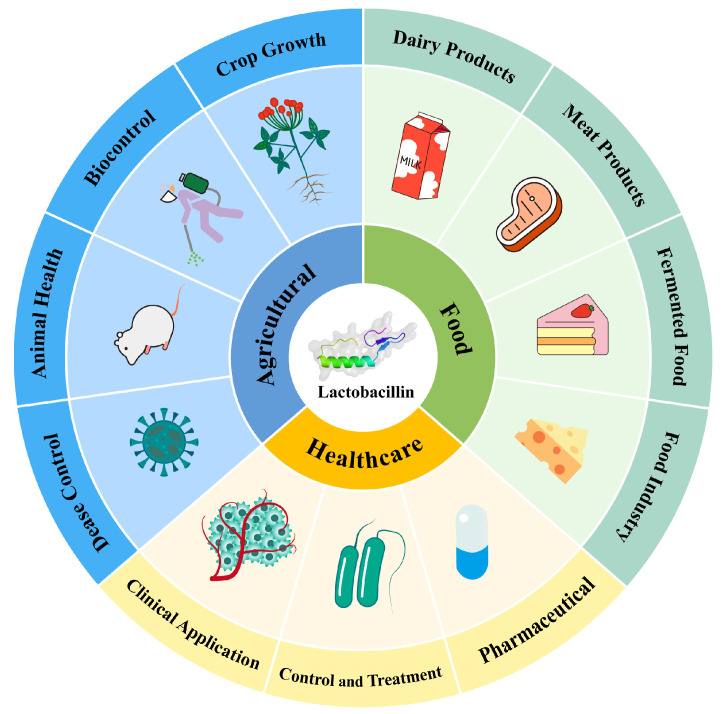
Application of LAB bacteriocin in various industries.

Traditional methods in food preservation, agriculture, and medicine face significant challenges, including chemical residues, resistance, and environmental pollution [128]. LAB bacteriocins offer safer and more sustainable alternatives. In food preservation, they extend shelf life and enhance quality. In agriculture, they control plant diseases and promote animal health. In medicine, they combat resistant pathogens and bolster immune responses. Figure 4 illustrates the applications and advantages of bacteriocins in these areas.

### 6.1. Food Preservation and Antimicrobial Packaging

Food is the most basic substance on which people depend for their survival. In recent years, the risks to human health posed by the use of chemical preservatives in food have become increasingly well known. As a result, there is a growing need to find new safe preservatives and methods of preservation [135]. LAB bacteriocins have been utilized in traditional food processing for over 50 years and have been recognized as Generally Regarded as Safe (GRAS) by the U.S. Food and Drug Administration (FDA) [135]. As natural preservatives, LAB bacteriocins can extend food shelf life and improve safety and quality. They are utilized in fermented dairy products as well as in antimicrobial packaging and biodegradable materials [17].

The application of LAB bacteriocins in food preservation overcomes significant limitations of traditional methods. They offer a natural and effective way to extend shelf life, enhance food safety, and reduce chemical usage. Nisin functions as a functional additive, enhancing the nutritional value and health benefits of food. Nisin has been shown to have no significant effect on Vero cells at concentrations below 11.4 μg/mL after 48 h of exposure. Additionally, it exhibits no notable toxicity to vaginal epithelial cells at a concentration of 318 μg/mL [136]. These findings highlight its excellent biosafety profile, alongside its ability to maintain food freshness and inhibit the growth of food spoilage microorganisms. Extensive research has been conducted on the application of nisin in food products [137]. Oladunjoye et al. demonstrated that treating fresh-cut tomatoes with 5000 IU/mL of nisin significantly inhibited the growth of *Listeria monocytogenes* [138]. Lee et al. treated beef jerky with 500 IU/g of nisin after 3 days of storage, finding that *Bacillus cereus* growth only began after 21 days. This treatment effectively prolonged shelf life and inhibited microbial growth [139]. Gharsallaoui et al. incorporated 2.5 to 12.5 mg/kg of nisin into vacuum-packed Korean seasoned beef and found that it strongly inhibited the growth of Bacillus subtilis [140]. There is also considerable research on other LAB bacteriocins in food. For example, enterocin AS-48 has been studied in egg liquid, targeting *Bacillus cereus* and *Staphylococcus aureus*. The minimum inhibitory concentrations at 4 °C and 28 °C were found to be 5 μg/mL and 0.05 μg/mL, respectively. Furthermore, electron microscopy revealed a synergistic effect between enterocin AS-48 and lysozyme [141]. Additionally, studies have found that pediocin PA-1/AcH applied in meat products inhibits the growth of *Listeria monocytogenes* more effectively than nisin, without affecting the growth of other bacteria [142]. Zhao et al. developed biodegradable polysaccharide substrates combined with natural antimicrobials for sustainable food packaging [17]. In meat products, pediocin demonstrated excellent antimicrobial effects, preventing contamination by *Staphylococcus aureus* and *Listeria* while maintaining activity under low-temperature conditions, making it suitable for various meat product preservation methods [26]. Narrow-spectrum bacteriocins, such as lactococcin 3147 and enterocin AS-48, have been used to selectively inhibit high-risk bacteria. Scannell et al. used immobilized *Lactococcus lactis* protein 3147 for in situ protection of cultures, resulting in a reduction in Staphylococcus aureus viable counts of approximately 1.5 log units in cheese and 2.8 log units in ham [143]. Enterocin TJUQ1, a novel class II bacteriocin, is effective against foodborne pathogens such as *Staphylococcus aureus*, *Listeria monocytogenes*, *Escherichia coli*, and *Salmonella*. The MIC against *Listeria monocytogenes* CMCC 1595 was found to be 5.26 ± 0.24 μg/mL [11]. Additionally, LAB bacteriocins have potential as food additives to enhance food functionality. LAB strains in traditional fermented dairy products, such as Bulgarian yogurt and feta cheese, contribute to microbial diversity, promoting digestion and nutrient absorption [144]. The diverse applications of bacteriocins—from fermented products to antimicrobial packaging—underscore their potential to revolutionize food preservation.

### 6.2. Agricultural Applications

The use of chemical pesticides and antibiotics in agriculture has led to significant issues, including environmental pollution, antibiotic residues, and the development of resistant pathogens [145]. These problems necessitate the exploration of natural and sustainable alternatives. LAB bacteriocins show promise in disease control, animal health management, and environmental protection, offering eco-friendly solutions that reduce reliance on synthetic chemicals and antibiotics [146].

In agriculture, LAB bacteriocins have shown significant effectiveness in disease control and animal health management. For plant disease control, LAB bacteriocins inhibit various pathogenic bacteria, including *Ralstonia solanacearum* and *Pseudomonas syringae*. They achieve this by inducing systemic resistance and competing for ecological niches, which reduces disease incidence [146]. Moreover, Gerez et al. found that certain LAB bacteriocins also inhibited plant pathogenic fungi, aiding in the control of fungal diseases such as powdery mildew and downy mildew [147]. In freshwater environments, *Streptococcus* and *Lactococcus* are considered pathogenic bacteria in aquaculture. Gatesoupe reported that adding probiotics such as *Carnobacterium* and *Enterococcus* to fish feed effectively stimulated the fish immune system and inhibited the proliferation of pathogens like *Streptococcus* and *Lactococcus* [148]. These bacteria are major pathogens in freshwater fish and play a critical role in the pathogenic dynamics of the aquatic microbiome. Wyszyńska investigated the use of LAB as an oral vaccine delivery system to control *Campylobacter* infections in chickens, which improved poultry health [149]. LAB adhere to intestinal epithelial cells, effectively inhibiting pathogens such as *Salmonella* and *E. coli*, and preventing enteric infections [150]. Millette et al. found that LAB bacteriocins, by inhibiting harmful bacteria, improved the intestinal microbial environment, promoted nutrient absorption, and enhanced animal growth rates [151]. As natural antimicrobials, LAB bacteriocins reduce the need for antibiotics, thereby lowering antibiotic residues and resistance issues, and supporting long-term disease management in animals [63]. In biocontrol and environmental protection, LAB bacteriocins reduce the need for chemical pesticides, decrease soil and water pollution, and contribute to environmental protection. Studies showed that LAB (such as *Lacticaseibacillus*, *Lactococcus*, and *Enterococcus*) could replace some herbicides, reducing environmental pollution and pesticide residues [152]. Due to their unique mechanisms of action, bacteriocins do not easily induce resistance in pathogens, thereby effectively addressing pesticide resistance issues [153]. LAB bacteriocins target harmful soil pathogens such as *Listeria*, *Actinomyces*, *Proteus*, and *Bacteroides*. Additionally, they foster beneficial microbial growth, enhance the soil microbial environment, improve soil fertility, and promote crop growth [154,155]. LAB bacteriocins offer an eco-friendly solution to agricultural challenges, overcoming the limitations of chemical pesticides and antibiotics. They effectively control plant and animal pathogens, enhance soil fertility, and promote plant growth while minimizing environmental impact.

### 6.3. Medical Applications

The widespread use of antibiotics in medicine has led to the emergence of resistant pathogens, making infections increasingly difficult to treat. Additionally, antibiotics can disrupt the human microbiota, leading to various health issues. LAB bacteriocins present promising alternatives or supplements to antibiotics. Their unique mechanisms minimize the risk of resistance development and selectively target pathogens without harming beneficial bacteria [156]. LAB bacteriocins hold significant potential in disease treatment and prevention. Notable examples include nisin Z, nisin A variants, and gallidermin, which are known to enhance immune responses [157]. Research suggests that combining bacteriocins with *Pseudomonas aeruginosa* lipopolysaccharides enhances innate immune responses without triggering septic reactions [158,159]. LAB bacteriocins, with their distinct mechanisms compared to antibiotics, are less likely to induce resistance, making them promising alternatives or supplements. Studies demonstrate that bacteriocins effectively inhibit a range of resistant pathogens, including Methicillin-resistant *Staphylococcus aureus* (MRSA), Vancomycin-resistant *Enterococcus* (VRE), and *Clostridioides difficile* infections (CDI) [160,161]. For antibiotic-resistant Gram-negative pathogens, studies have shown that *Acidophilus* STRAIN 52 and *Limosilactobacillus fermentum* STRAIN 45 inhibit Cephalosporin-resistant *Escherichia coli* strains with a 100% inhibition rate [162]. In a cell lysis experiment, the addition of 6400 Au/mL of bacteriocin inhibited the growth activity of *Pseudomonas aeruginosa*, reducing its activity from 8.7 × 10^6^ CFU/mL to 1.3 × 10^7^ CFU/mL [163]. Acidocin exhibited a moderate antibacterial effect against drug-resistant *Pseudomonas aeruginosa*, with an inhibition zone of 15.00 mm [164]. Lü et al. isolated Lactocin MXJ 32A from the *Loigolactobacillus coryniformis* MXJ 32 strain, which strongly inhibited the growth of *Salmonella* (inhibition zone > 20 mm). The antibacterial activity against *Salmonella* strains 87T4, 1006D, 36T, 557D, and 798D was measured at 4112.4, 3812.1, 4288.4, 4422.5, and 3770.0 AU/mL, respectively [165]. For antibiotic-resistant Gram-positive pathogens, the bacteriocin MXJ 32A produced by *Loigolactobacillus coryniformis* showed an inhibition zone of 23 mm against *Staphylococcus aureus*, with an antibacterial activity of 4650.0 AU/mL [165]. For antibiotic-resistant Gram-positive pathogens, the bacteriocin MXJ 32A produced by *Loigolactobacillus coryniformis* showed an inhibition zone of 23 mm against *Staphylococcus aureus*, with an antibacterial activity of 4650.0 AU/mL [166]. For antibiotic-resistant Gram-positive pathogens, the bacteriocin MXJ 32A produced by *Loigolactobacillus coryniformis* showed an inhibition zone of 23 mm against *Staphylococcus aureus*, with an antibacterial activity of 4650.0 AU/mL [145,167]. Additionally, bacteriocins EF478, BLIS, and Gallocin D demonstrated antibacterial activities against VRE with values of 320.0 AU/mL, 3000.0 AU/mL, and 1.56 µg/mL, respectively [168,169].

In clinical applications, LAB bacteriocins have garnered significant attention. LAB bacteriocins and their probiotic active cell substances exert numerous beneficial effects in the gastrointestinal tract, preventing pathogen adhesion, establishment, and replication through multiple antimicrobial mechanisms [170]. Products containing nisin effectively inhibit oral pathogens, helping to prevent gingivitis and oral ulcers [171,172]. For instance, *Limosilactobacillus fermentum* HV6b MTCC produces an Lla-class bacteriocin peptide, HV6b, which effectively inhibits various pathogens associated with human vaginal infections, including *Bacteroides*, *Gardnerella vaginalis*, *Staphylococcus*, and *Streptococcus* in vitro [173]. Additionally, nisin shows potential for targeted cancer therapy [174]. Advances in nanodrug delivery systems and genetic engineering have positioned bacterium-based diagnostic and therapeutic delivery systems as a major research focus in chemical biology [98,175]. LAB bacteriocins can target cell membranes to avoid immune responses and induce cell death directly through pore formation, thereby reducing systemic drug toxicity. Researchers have developed natural biomimetic delivery systems using these bacteriocins to address antibiotic resistance. LAB also exhibit antitumor effects by inhibiting mutagenic activity and reducing the production of carcinogens and associated enzymes, demonstrating potential for cancer prevention and treatment [176,177,178]. Studies have shown that specific cellular components of LAB strains can induce strong adjuvant effects, regulate cell-mediated immune responses, activate the reticuloendothelial system, enhance cytokine pathways, and modulate interleukins and tumor necrosis factors [179,180,181,182]. For example, plantaricin effectively inhibits pathogens causing skin infections, thereby accelerating wound healing through its antimicrobial properties. Reuterin exhibits broad-spectrum antimicrobial activity, effectively targeting a wide range of pathogenic bacteria in the gastrointestinal tract. Clinical studies have evaluated its efficacy in modulating gut microbiota, reducing gastrointestinal infections, and alleviating inflammatory bowel disease symptoms. The probiotic potential of *Limosilactobacillus* and its reuterin production underscore its role in maintaining gut health and preventing gastrointestinal disorders. Additionally, LAB bacteriocins can reduce reliance on synthetic antibiotics and help manage emerging immunocompromised populations [183].

LAB bacteriocins offer a viable solution to challenges posed by antibiotic resistance and human microbiota disruption. Their unique mechanisms and targeted action enable the effective treatment and prevention of infections. Additionally, LAB bacteriocins enhance immune responses and provide therapeutic benefits in cancer and gastrointestinal disorders. Integrating bacteriocins into clinical applications reduces reliance on synthetic antibiotics, mitigates resistance, and offers safer, more effective treatments. The potential antiviral properties of LAB bacteriocins have garnered significant attention from researchers. For instance, a peptide produced by *Enterococcus mundtii* ST4V has been demonstrated to exhibit notable antiviral activity [73], effectively inactivating multiple strains of herpes simplex virus (HSV), poliovirus, and an attenuated measles virus strain. Similarly, Todorov et al. investigated a pediocin-like peptide produced by *Enterococcus faecium* ST5Ha, which showed a strong inhibitory effect against herpes simplex virus type 1 (HSV-1) in vitro [184]. Despite these findings, the precise mechanisms through which bacteriocins act against viruses remain unclear. Wachsman et al. proposed that these mechanisms may involve blocking viral receptor binding sites or inhibiting viral replication [185].

The activity of LAB bacteriocins observed during in vitro studies offers exciting new insights into the development of cancer treatment strategies. Chiu et al. reported that certain factors produced by *Lactobacillus rhamnosus* and *Lacticaseibacillus* significantly induced apoptosis pathways in monocytic leukemia cell lines, effectively suppressing cancer cell proliferation [186]. Additionally, an experimental study in mice demonstrated that feeding *Lacticaseibacillus helveticus* R389 delayed tumor progression [187]. Further investigations revealed that this effect might be attributed to reduced levels of pro-inflammatory cytokines such as IL-6 [187]. These groundbreaking findings not only highlight the potential of bacteriocins in the fight against cancer but also emphasize their value as promising adjuvants in cancer therapy. Their combined immunomodulatory and pro-apoptotic properties open new avenues for integrating bacteriocins into advanced cancer treatment strategies [188].

## 7. Future Directions and Prospects

LAB bacteriocins are widely acknowledged for their safety and high antimicrobial efficiency, making them valuable as natural food preservatives [189]. Their applications extend to preserving animal-derived foods, with commercial examples like nisin and pediocin successfully inhibiting spoilage bacteria and pathogens in several countries [190]. Additionally, bacteriocins such as Enterocin AS-48 [191], Bovicin HC5 [192], Enterocin 416K1 [61], pediocin, and Bifidin C6165 [193] have demonstrated potential in extending the shelf life of fruit-based products. Despite substantial advancements in LAB bacteriocin research, their clinical and industrial applications encounter persistent challenges. Key obstacles include low production efficiency, high purification costs, and stringent regulatory requirements [20]. Current methods, such as chemical synthesis and heterologous expression, often yield low quantities and inadequate purity, undermining economic feasibility and application scalability [194,195]. The incomplete understanding of bacteriocin mechanisms represents another significant barrier to their practical application [195,196]. This knowledge gap delays industrialization and raises the standards for further development. Bacteriocin stability and activity can be compromised under challenging environmental conditions, such as high temperatures, extreme pH levels, or complex food matrices. For example, nisin stability decreases in acidic environments, limiting its application in certain food preservation scenarios. These limitations restrict their use in specific industrial processes and highlight the need for stability-enhancing modifications. Moreover, their relatively weaker inhibitory effects on Gram-negative bacteria fail to meet the demands for broad-spectrum antimicrobial applications. These limitations highlight the importance of developing more stable and active bacteriocins or their derivatives as a critical pathway to overcoming application bottlenecks.

In both agriculture and medicine, LAB bacteriocins, as natural antimicrobial agents, have shown great potential for various applications. Particularly in agriculture, LAB bacteriocins can be used as biopesticides and plant growth promoters to control crop diseases and enhance plant growth. However, the in vitro activity of LAB bacteriocins does not always translate effectively in complex agricultural environments. LAB are ubiquitous members of many plant microbiomes, but there have been no reports on how LAB interact with hosts to exert antimicrobial effects. Moreover, LAB bacteriocins exhibit relatively weak inhibitory activity against plant pathogenic fungi and bacteria. For instance, in soil and the rhizosphere microbial communities of plants, the antimicrobial efficacy of bacteriocins may be influenced by factors such as soil composition, environmental temperature, and humidity [197,198]. LAB bacteriocins are present in very low concentrations in organic agricultural soils. Currently, they exert antimicrobial effects primarily through exogenous application, such as spraying or coating, rather than through natural production in the soil [199]. Research on these environmental factors is still in its early stages. Future studies should include more field trials to evaluate the practical application of LAB bacteriocins in different agricultural contexts.

The mechanisms of LAB bacteriocins in clinical applications are complex. Compared to conventional antibiotics, the clinical potential of LAB bacteriocins is mainly limited by their narrow antimicrobial spectrum and inability to directly target bacterial infection sites. Additionally, their presumed antigenicity and potential bio-toxicity further restrict their use. In the medical field, LAB, as intestinal probiotics, contribute to gut health by preventing and treating related disorders, thus supporting the overall health of the host. Research has shown that lactic acid bacteriocins can impact gut health and immune system function. This occurs either by increasing the proportion of beneficial bacteria in the gut microbiota or by directly influencing host metabolism [200,201]. Their inhibitory effects on antibiotic-resistant strains, particularly Gram-positive bacteria, have been partially validated. However, the absorption, distribution, metabolism, and therapeutic effects of bacteriocins in the human body remain insufficiently understood. Further exploration through in vivo experiments and preclinical studies is essential to elucidate their activity and potential therapeutic value. Despite evidence from animal studies demonstrating the ability of LAB bacteriocins to inhibit resistant bacterial strains, their long-term impact on the human microbiome remains unclear. Therefore, medical applications must carefully assess their long-term effects on human health, particularly on the immune system and microbiome. To minimize potential risks, future research should focus on the safety assessment of LAB bacteriocins and conduct long-term preclinical and clinical trials.

To address these challenges, the development of efficient and cost-effective separation and purification technologies is critical. Such advancements could not only reduce production costs but also expand the application fields of bacteriocins [195]. For instance, key technological breakthroughs may be achieved through protein engineering, genetic modification, or the chemical synthesis of novel antimicrobial peptides [202,203]. These technological advancements could promote growth in the food industry and pave the way for broader applications in animal feed, organic fertilizers, environmental protection, and personal care products [204,205]. While genetic and metabolic engineering technologies can enhance the production yield of bacteriocins to some extent, their adoption remains constrained by cost pressures and regulatory limitations, particularly in food and pharmaceutical sectors where stricter safety standards are required. These challenges suggest that, beyond technological breakthroughs, supportive regulations and policies are equally essential. From a future development perspective, the sustained progress of LAB bacteriocins requires dual drivers of technological innovation and regulatory support. On the one hand, the application of modern biotechnologies, such as heterologous expression systems, intelligent bioreactors, and metabolic engineering, will significantly improve production efficiency and reduce costs. On the other hand, close collaboration with regulatory agencies through systematic safety and efficacy validation studies can provide guarantees for the marketization of bacteriocin products. Simultaneously, their application fields are expected to expand further, such as combining with biodegradable packaging materials in the food industry, developing composite biopesticides in agriculture, and exploring personalized therapeutic strategies for specific diseases in the medical field.

Future development priorities should focus on several aspects. First, enhancement of high-throughput screening technologies should be prioritized to discover new bacteriocins with higher antimicrobial activity and broader-spectrum effects. Predictive models based on computational biology and artificial intelligence can significantly accelerate this process, substantially shortening research cycles. Additionally, leveraging synthetic biology to design and optimize bacteriocin structures can further enhance their stability and functionality. For example, modifications to amino acid sequences could lead to the development of bacteriocins that are more heat-resistant, pH-stable, or adaptable to complex environments, offering solutions for specialized processing environments and complex applications.

Second, in terms of production processes, exploring the combination of continuous fermentation technologies and microbial co-culture systems could improve production efficiency and reduce resource consumption. By precisely controlling fermentation conditions and metabolic pathways, production yields could be significantly increased while reducing the accumulation of by-products, enhancing the overall sustainability of production processes. Additionally, purification techniques based on green chemistry and environmentally friendly processes, such as membrane separation and nanotechnology, could enable efficient and cost-effective bacteriocin extraction and purification.

Third, the multifunctional applications of LAB bacteriocins merit further exploration. For example, in food safety, antimicrobial packaging materials with real-time monitoring capabilities could be developed using Internet of Things (IoT) technologies to dynamically monitor and control bacterial contamination. In agriculture, integrated solutions combining bacteriocins with eco-friendly pesticides could minimize the use of chemical pesticides while improving crop yield and quality. In medicine, by integrating with precision medicine and gene therapy technologies, bacteriocins could be explored for applications in specific pathogenic infections, tumor immunotherapy, and even gut microbiota regulation.

Moreover, the role of bacteriocins in ecological balance and environmental protection also warrants attention. Through ecological engineering, bacteriocins could be used to regulate environmental microbial communities to remediate polluted soils or improve water quality. Additionally, combining bacteriocins with biodegradable materials could lead to the development of sustainable, eco-friendly products to support carbon neutrality goals.

Finally, international collaboration and policy development are crucial for driving the advancement of bacteriocins. Strengthening international technological cooperation and data sharing could accelerate the promotion of bacteriocin applications worldwide. Simultaneously, refining regulatory frameworks for bacteriocin-related products could pave the way for their commercialization on a global scale.

Through multidimensional technological innovations and cross-disciplinary resource integration, LAB bacteriocins are poised to overcome existing limitations and play a larger role in food safety, green agriculture, environmental protection, and precision medicine. Looking ahead, LAB bacteriocins are not only tools to address current challenges but also potential engines driving the bioeconomy and sustainable development.

## 8. Conclusions

In conclusion, although lactic acid bacteriocins have advanced as natural food preservatives, their broader application still faces several challenges. Addressing high purification costs, optimizing synthesis methods, exploring new applications, and securing regulatory approval are essential steps. With technological innovation and regulatory support, the future prospects for bacteriocins are promising, offering new opportunities across various industries.
